# Religious beliefs and practices toward HPV vaccine acceptance in Islamic countries: A scoping review

**DOI:** 10.1371/journal.pone.0309597

**Published:** 2024-08-29

**Authors:** Sezer Kisa, Adnan Kisa

**Affiliations:** 1 Faculty of Health Sciences, Department of Nursing and Health Promotion, Oslo Metropolitan University, Oslo, Norway; 2 School of Health Sciences, Kristiania University College, Oslo, Norway; 3 Department of International Health and Sustainable Development, Tulane University, New Orleans, United States of America; University of Energy and Natural Resources, GHANA

## Abstract

**Background:**

Despite the availability of effective HPV vaccines, their acceptance in Islamic countries is often influenced by religious beliefs, practices, and misconceptions.

**Objective:**

This review aimed to identify the current literature on the religious beliefs and any misconceptions toward HPV vaccine acceptance within the Organisation of Islamic Cooperation (OIC) countries.

**Method:**

Using key terms, a systematic search in MEDLINE/PubMed, Embase, and CINAHL yielded 23 studies that met the inclusion and exclusion criteria. The scope of this review included all research articles published in English until October 31, 2023. A form based on the aim of the study was developed and used to extract the data.

**Results:**

The review highlights the complexity of the relationship between religious beliefs and HPV vaccine uptake. The findings reveal significant objections among a number of Muslims. Some of them believe vaccines lead to infertility and sexual promiscuity, defy religious norms, are a sneaky way to inject good Muslims with haram ingredients, and are an abandonment of righteous principles in general.

**Conclusions:**

Vaccine hesitancy is a result of doubts regarding the vaccine’s safety, necessity, and compatibility with religious beliefs. It is recommended to encourage HPV vaccine uptake in Islamic countries by using public health strategies that adopt a holistic approach that incorporates religious, cultural, and social aspects.

## Introduction

Human papillomavirus (HPV) is a sexually transmitted infection commonly seen in low- and middle-income countries [1,2]. The global spread of HPV remains a significant public health challenge. It is the leading cause of cervical cancer, which is the fourth most significant cause of cancer-related death among women around the world. It is also responsible for cancers in the anus, vulva, vagina, penis, head, and neck [1,3]. Studies indicate a rising trend of HPV infection in young women who engage in early sexual activities [3–5]. The underlying risk factors for HPV infection include low socioeconomic status, other sexually transmitted agents, multiple sexual partners, early marriage, early onset of sexual activity, immunosuppression, more permissive sexual attitudes among the younger population, and unprotected sex [2,4].

Preventive public health strategies, including cervical screening and vaccination, protect against the most harmful types of the virus (types 16 and 18) [5]. The World Health Organization recommends vaccinating girls aged 9–14 who have not yet initiated sexual activity and those up to age 25 who have not been previously vaccinated [5]. The HPV vaccine is widely recognized for its efficacy in preventing cervical cancer, which is primarily caused by high-risk HPV types such as HPV-16 and HPV-18 [3,6,7]. It also reduces the incidence of genital warts, which are caused by low-risk HPV types such as HPV-6 and HPV-11 [8,9]. These benefits can influence acceptance and attitudes towards the vaccine, as some individuals may view it as a cancer prevention tool, while others may value its ability to reduce the morbidity associated with sexually transmitted infections. The global adoption of these vaccines faces challenges, such as: lack of recommendation from a physician [10,11]; family acceptance or parental opposition/ignorance [11,12]; fear of side effects; fearing being too young for the vaccine [13]; lack of knowledge about HPV transmission, cervical cancer, and vaccines [14,15]; skepticism about the vaccine’s content, safety, and effectiveness [13]; and concerns about the costs of vaccination [5,15–17].

It is well-established in the literature that religious beliefs have a profound impact on an individual’s decision-making and health-related behaviors, including sexual health [18–20]. Mouallif et al. (2014) note that leaving health outcomes to God’s will is a common belief [21], while another study highlights the preference for faith healing and traditional medicine over orthodox methods [22]. Additionally, adherence to religious principles, such as abstaining from premarital sex or believing that religiously-based circumcision reduces HPV prevalence, play a role in shaping one’s health-related choices [17,23]. Religious beliefs, derived from religious teachings, guide moral and ethical decisions and influence behavior. Dietary rules that distinguish what is permissible from what is forbidden also play a role in guiding behavior. Religious beliefs may encompass traditional healing practices and interpretations from religious authorities, who set community standards and shape ethical decisions [24–27]. The influence of religion goes beyond personal beliefs. It shapes attitudes toward preventive health measures such as vaccination [12,17,22]. It has been proven that there is a strong relationship between religious beliefs and vaccine acceptance, including vaccine decision-making for sexually transmitted infections such as HPV [12,21,28,29]. A study in Saudi Arabia found that religious objections accounted for 30% of opposition to the vaccine [28]. Another study noted the role of religious leaders in shaping vaccine uptake within African communities [30]. Some people may perceive vaccines as consistent with their religious principles and view vaccines as a means of preserving health in line with divine will [31]. Conversely, some religious perspectives may lead to hesitancy or resistance due to concerns about the vaccine’s content (e.g., claims that a vaccine was processed from pig’s blood), moral implications, or perceived conflicts with sacred teachings [12,32].

Islamic countries exhibit unique socio-cultural dynamics in which religious beliefs and practices play an important role in shaping social norms. It is commanded in Islam to abstain from sex until after marriage. Since HPV is sexually transmitted, some parents may believe that vaccinating their daughters is unnecessary and immoral because it may encourage sexual activity at an early age [12]. Pelčić and colleagues (2016) found that, despite the absence of any taboo against vaccination and a general alignment of religion with vaccines and public health, there has been a rise in vaccine refusal attributed to religious objections [33]. Furthermore, recent events in Indonesia have highlighted the impact of religious rulings on immunization rates, as Islamic clerics declared a measles-rubella vaccine containing pork components as impure, leading to a significant decline in vaccine coverage [34]. Additionally, a multi-country analysis conducted in sub-Saharan African countries found that in several nations, including those with significant Muslim populations, there were lower levels of vaccine coverage among Muslim communities compared to Christian ones [35]. This trend is frequently linked to individual parents or religious leaders opposing vaccination and providing questionable interpretations of religious teachings. Despite the abundance of studies on the safety of the HPV vaccine, there is little research on how religious practices influence vaccine acceptance in these communities. Understanding the full spectrum of the vaccine’s benefits is crucial in shaping public perception and acceptance [9,36] and is particularly relevant in the context of Islamic countries, where cultural and religious beliefs significantly influence health behaviors. This lack of understanding hinders the development of targeted interventions to address barriers arising from religious attitudes. Research has focused on knowledge about HPV vaccines themselves, leaving a gap in understanding religious beliefs about vaccines and their acceptance. Therefore, this review is guided by the following research questions:

1. Does religion influence HPV vaccine acceptance in the Organisation of Islamic Cooperation (OIC) countries?
2. What religious beliefs and practices potentially forbid the HPV vaccine in the OIC countries?
3. What objections and misconceptions against the HPV vaccine are found in the OIC countries?

## Method

This study was designed as a scoping review, following the methodology described by the Joanna Briggs Institute, to systematically map the existing literature on religious beliefs, practices, and misconceptions regarding HPV vaccine acceptability, and to identify any research gaps. This method is particularly effective for investigating emerging research domains and generating practical evidence [37]. Our methodology was guided by the principles developed by Arksey and O’Malley (2005) [38]. The inclusive nature of the scoping review allowed for an examination of a wide range of literature, including but not limited to primary research studies, systematic reviews, meta-analyses, correspondence, guidelines, and various online resources. We started by enlisting the aid of a librarian to identify the aim and research questions and to develop the research strategy.

Following JBI’s guidelines, a three-stage search strategy was formulated. The first stage entailed searching Google Scholar. In the second stage, key terms from titles, abstracts, and index lists of articles were identified by one of the reviewers (AK), and the MEDLINE/PubMed, Embase, and CINAHL databases were searched. Database-specific vocabulary focused on “HPV vaccine” OR “HPV Vaccination” in conjunction with the names of the countries within the OIC [39]. These countries are Algeria, Benin, Burkina Faso, Cameroon, Chad, Comoros, Djibouti, Egypt, Gabon, Gambia, Guinea, Guinea-Bissau, Ivory Coast, Libya, Mali, Mauritania, Morocco, Mozambique, Niger, Nigeria, Senegal, Sierra Leone, Somalia, Sudan, Togo, Tunisia, Uganda, Afghanistan, Azerbaijan, Bahrain, Bangladesh, Brunei, Indonesia, Iran, Iraq, Jordan, Kazakhstan, Kuwait, Kyrgyzstan, Lebanon, Malaysia, Maldives, Oman, Pakistan, Palestine, Qatar, Saudi Arabia, Syria, Tajikistan, Turkey, Turkmenistan, United Arab Emirates (UAE), Uzbekistan, and Yemen (Table 1). The last stage of the research was expanded to include tracing references from relevant studies and examining gray literature to identify additional studies.

**Table 1:** Example search strategy (MEDLINE/PubMed).

Search Item	Search Component	Search Terms
#1	HPV Vaccine	"HPV vaccine" OR "HPV Vaccination"
#2	Countries	Algeria OR Benin OR Burkina Faso OR Cameroon OR Chad OR Comoros OR Djibouti OR Egypt OR Gabon OR Gambia OR Guinea OR Guinea-Bissau OR Ivory Coast OR Libya OR Mali OR Mauritania OR Morocco OR Mozambique OR Niger OR Nigeria OR Senegal OR Sierra Leone OR Somalia OR Sudan OR Togo OR Tunisia OR Uganda OR Afghanistan OR Azerbaijan OR Bahrain OR Bangladesh OR Brunei OR Indonesia OR Iran OR Iraq OR Jordan OR Kazakhstan OR Kuwait OR Kyrgyzstan OR Lebanon OR Malaysia OR Maldives OR Oman OR Pakistan OR Palestine OR Qatar OR Saudi Arabia OR Syria OR Tajikistan OR Turkey OR Turkmenistan OR United Arab Emirates OR Uzbekistan OR Yemen
#3	#1 AND #2	("HPV vaccine" OR "HPV Vaccination") AND (Algeria OR Benin OR Burkina Faso OR Cameroon OR Chad OR Comoros OR Djibouti OR Egypt OR Gabon OR Gambia OR Guinea OR Guinea-Bissau OR Ivory Coast OR Libya OR Mali OR Mauritania OR Morocco OR Mozambique OR Niger OR Nigeria OR Senegal OR Sierra Leone OR Somalia OR Sudan OR Togo OR Tunisia OR Uganda OR Afghanistan OR Azerbaijan OR Bahrain OR Bangladesh OR Brunei OR Indonesia OR Iran OR Iraq OR Jordan OR Kazakhstan OR Kuwait OR Kyrgyzstan OR Lebanon OR Malaysia OR Maldives OR Oman OR Pakistan OR Palestine OR Qatar OR Saudi Arabia OR Syria OR Tajikistan OR Turkey OR Turkmenistan OR United Arab Emirates OR Uzbekistan OR Yemen)

**Fig 1:**
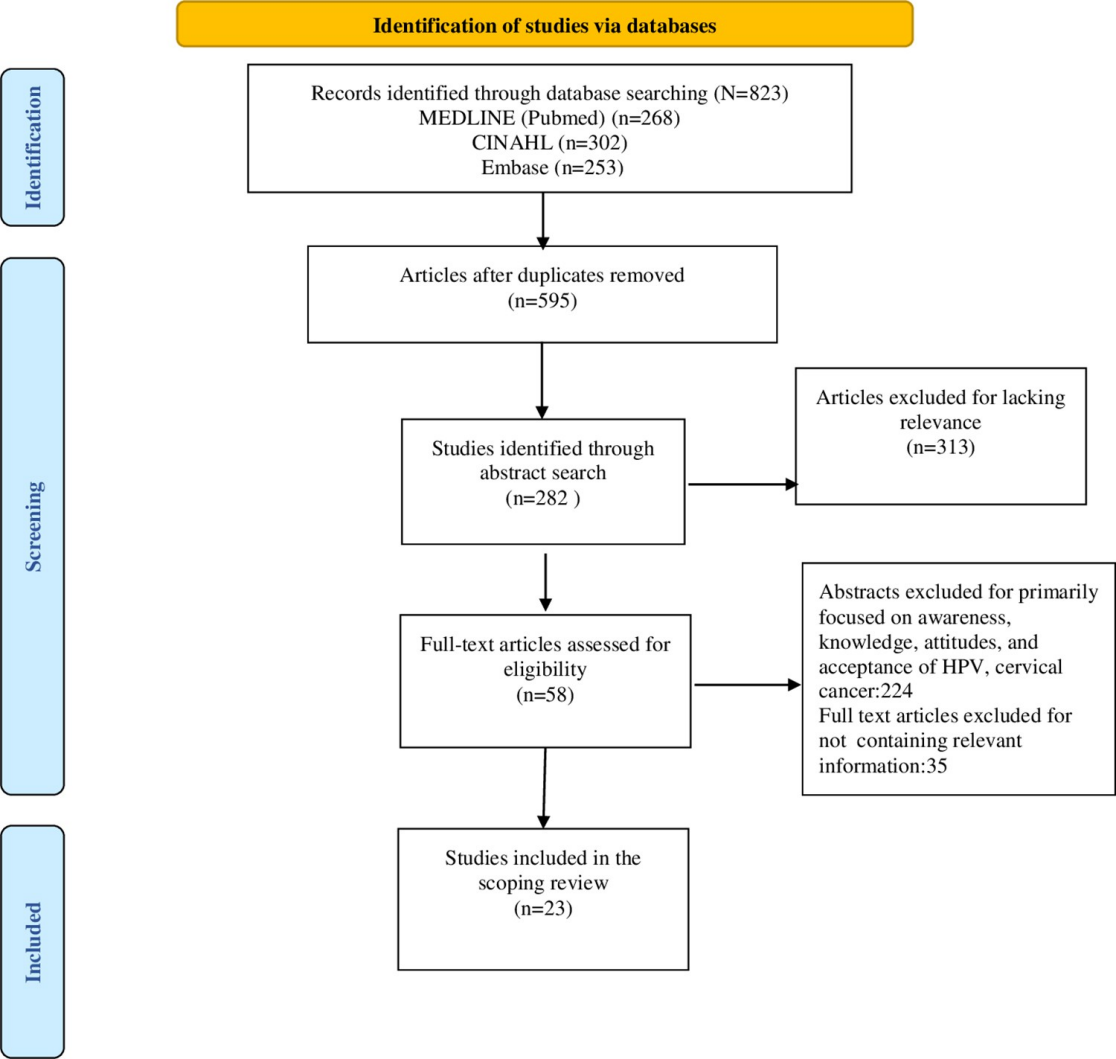
PRISMA flowchart.

Following a systematic search based on predefined inclusion and exclusion criteria, relevant studies were identified and imported into EndNote (version 21). We did not conduct a quality assessment since it’s not recommended in scoping reviews [38]. The scope of this review included all research articles published in English until October 31, 2023. Sources for this review were carefully selected, focusing on databases rich in medical and public health literature. The initial phase involved database searches conducted by one reviewer (AK), with subsequent removal of duplicate records. Two reviewers (AK and SK) independently assessed titles and abstracts, followed by full-text reviews and data extraction. Discrepancies between the reviewers were resolved through discussion. Articles that did not meet the eligibility criteria to address the research questions were excluded. Excluded studies were those that focused on vaccine acceptance barriers beyond religious beliefs, practices, and misconceptions; were published as dissertations, reviews, conference abstracts, editorials, opinion pieces, or came from non-peer-reviewed sources; were published in languages other than English; or which primarily investigated HPV knowledge and awareness. The PRISMA flow chart in Fig 1 presents detailed information on the exclusion process.

Data were extracted into an extraction form designed by a single reviewer (SK) in alignment with the research questions. The form included data pertinent to the characteristics of the studies (author(s), publication year, journal, study aims, research question, population, method, sample size, outcomes, and contextual details) and information related to the religious aspects of HPV vaccine acceptance. Both reviewers endorsed the extraction form, deliberated on each element to be captured, and resolved any conflicts. The findings were organized into two primary categories that addressed the research questions regarding religious beliefs and practices, and objections and misconceptions.

## Results

### Study characteristics

For this scoping review, a comprehensive examination of 595 studies was conducted. From these, 282 studies were selected through their abstracts, and 23 articles were found eligible for review to answer the study’s research questions. These eligible studies represented a wide geographic range, with their distribution as follows: Gambia (1), Indonesia (4), Malaysia (2), Morocco (1), Nigeria (7), Pakistan (1), Uganda (2), Saudi Arabia (3), and the United Arab Emirates (2). Notably, all these countries are predominantly Sunni, which is the largest branch of Islam. Among the included articles, most were designed as cross-sectional studies and published in 2023 (six studies), followed by six studies in 2022 and three in 2021. The diverse sample populations provided a comprehensive overview of the issue across different demographics, enhancing the generalizability of the findings. The variety in settings, from urban centers to rural communities, allowed for a complete understanding of how different environments influence religious beliefs and practices.

The studies examined various levels of awareness and acceptance of the HPV vaccine. A school-based HPV immunization program in Malaysia was found to be effective in a multicultural and religious society [40]. In Nigeria, healthcare professionals showed high awareness of HPV (91%) and the vaccine (44%), with male professionals significantly more likely than their female counterparts to approve the vaccine for their teenage daughters [41]. In Morocco, awareness and acceptability of the HPV vaccine were low, and were influenced by education, income, and religious beliefs, with mothers being less likely than fathers to accept the vaccine [21]. Among male university students in the UAE, knowledge of HPV was low (32%), with religious objections and concerns about vaccine safety being common [42]. In Nigeria, females demonstrated higher awareness of cervical cancer and were generally more receptive to HPV vaccination compared to males [22].

In Indonesia, a high willingness to receive the HPV vaccine was observed among university students, particularly females [43]. Knowledge levels about HPV and vaccine acceptability in Saudi Arabia showed no significant gender differences [28]. Cultural and safety concerns influenced vaccine acceptance among Emirati men [44]. Despite these insights, it is important to note that 13 out of the 23 reviewed studies did not include any gender-specific analyses. This indicates a gap in the literature regarding the differential impact of religious beliefs on HPV vaccine acceptance between males and females. However, exploring gender differences was not the primary aim of this review [40,42,44–54].

The results are summarized in Table 2.

**Table 2:** Summaries of studies on religious influence on HPV vaccination.

Country, Year, Author/s	Methodology	Aim	Concept	Outcome	Religious Beliefs and Practices	Religious Objections and Misconceptions
The Gambia, 2023, Wilson et al. [44]	Qualitative study In-depth interviews and focus group discussions 125 participants (73 interviews and 10 focus groups)	Investigate HPV vaccination acceptance and perceptions related to fertility and population control	HPV vaccine acceptance and perceptions related to fertility and population control	Concerns about infertility has hindered HPV vaccine acceptance despite high overall uptake. Females scored lower in baseline knowledge questions about HPV transmission and its gender-specific impact	Majority Muslim population; cultural and traditional beliefs influencing perceptions of family planning and vaccination	Beliefs about the vaccine being a birth control measure were common, reflecting a cultural perspective possibly influenced by religious beliefs about family planning and childbirth
Indonesia, 2021, Khatiwada et al. [43]	Cross-sectional Self-administered online questionnaire 433 students from Universitas Padjadjaran	Assess the knowledge, attitude, and willingness of university students toward HPV vaccination	Knowledge, attitude, and acceptability of HPV vaccination	A significant association between willingness to vaccinate and recommend vaccines to others High willingness (95.8%) to receive the HPV vaccine among female respondents	Religious and cultural factors played a role in vaccine acceptance among the participants	Rumors about vaccines containing haram substances (such as pig components) and not being natural
Indonesia, 2021, Wijayanti et al. [53]	Cross-sectional Questionnaire-based survey 680 parents from 33 primary schools in Jakarta	Examine parents’ beliefs, attitudes, and intentions regarding their daughters’ receipt of the HPV vaccine and analyze its uptake	Parents’ attitudes, beliefs, and uptake of the school-based HPV vaccination program	Most parents allowed vaccination for their daughters Attitudes, subjective norms, and perceived behavioral control were significant predictors of HPV vaccine uptake	Parents’ decisions regarding HPV vaccination for their daughters was significantly influenced by subjective norms, which may include religious and community influences	
Indonesia, 2022, Frianto et al. [47]	Cross-sectional Questionnaire-based survey 286 parents from West Java	Determine parental acceptance of HPV vaccination in districts with a high prevalence of cervical cancer in West Java, Indonesia, considering various factors, including household income	Parental acceptance of HPV vaccination	Health beliefs toward cervical cancer significantly correlated with HPV vaccination acceptance	Religion impacted their decision-making process A minority perceived either a positive or a negative religious impact on their decision-making	Religion was not perceived as a barrier to HPV vaccination
Indonesia, 2022, Winarto et al. [55]	Cross-sectional Online self-reported questionnaire 400 citizens from Jakarta	Investigate the association between knowledge, attitudes, practices, and socio-demographic influences related to HPV, cervical cancer, and vaccination among urban Indonesians	Knowledge, attitudes, and practices towards HPV, cervical cancer, and vaccination	Poor knowledge in individual aspects, but moderate overall knowledge, good overall attitude, and unsatisfying practices Women exhibited significantly better knowledge than men in attitudes and practices regarding HPV infection, cervical cancer, and HPV vaccination	Religious beliefs could play a role in their attitudes and practices regarding HPV, cervical cancer, and vaccination	
Malaysia, 2012,Ling et al. [40]	Survey Questionnaire 1500 teachers in urban secondary schools	Assess teachers’ knowledge and perception of HPV, cervical cancer, and HPV vaccine prior to a school-based vaccination program	Knowledge and perception of HPV and HPV vaccination	School-based HPV immunization program can be effectively implemented in a multicultural and religious society	Religion has a negative influence on HPV vaccination programs	Despite the concern that religion might be an issue in implementing the HPV vaccine in a predominantly Islamic society, the study showed that religiosity did not significantly reduce the likelihood of vaccination 95% of the respondents did not believe that vaccination would increase promiscuity, indicating that religious or moral objections were not major barriers to HPV vaccination acceptance
Malaysia, 2022, Wong et al. [54]	Cross-sectional Questionnaire 900 patients at a university teaching hospital, obstetric and gynecology clinics	Investigate HPV vaccination intention among married women and its associated factors	HPV vaccination intention among married women	High intention (74.5%) to vaccinate was influenced by spouse/partner’s consent, occupation, household income, and knowledge about HPV	Malay Muslim participants perceived need for their husbands’ approval for HPV vaccination reflects traditional and religious cultural norms	HPV vaccination was unnecessary in a marriage with proper religious upbringing
Morocco, 2014, Mouallif et al. [21]	Survey Questionnaire 852 parents at public and private health centers and clinics	Assess awareness of HPV and vaccine and factors associated with vaccine acceptability among parents	Parental acceptability of HPV vaccine for daughters	Very low awareness of HPV (4.7%) and vaccine (14.3%) Low vaccine acceptance Mothers were less likely to accept the vaccine than fathers Education, income, previous awareness of the vaccine and religious beliefs affected acceptance	Fatalism and belief that health outcomes are God’s will reduced acceptance of the HPV vaccine	A strong influence of religious beliefs or fatalistic views toward medical interventions on health decisions
Nigeria, 2014, Audu et al.[41]	Cross-sectional Questionnaire 610 healthcare professionals from three tertiary institutions	Assess healthcare professionals’ knowledge and perception of HPV and the vaccine and their willingness to vaccinate their teenage daughters	Awareness and perception of the HPV vaccine	91% knew of HPV 44% knew about the vaccine 81% approved the vaccine for their teenage daughters Male healthcare professionals were much more likely than female professionals to approve vaccination for their daughters		Acknowledged the significant role of religion in Nigerian society Religious objections or concerns did not significantly impact the perception of HPV vaccine among the healthcare professionals surveyed
Nigeria, 2018,Balogun & Omotade, [22]	Exploratory qualitative study Focus group discussions (20) and key informant interviews (4) 190 adolescents from five selected communities in the Ibadan, Nigeria Government Area	Explore interpretations of cervical cancer and views on HPV vaccination among adolescents in Ibadan	Cervical cancer, HPV vaccination	Causes of cervical cancer Attitudes of adolescents toward HPV vaccination Females demonstrated higher awareness of cervical cancer and were generally more receptive to HPV vaccination compared to males	Religious leaders, both Christian and Islamic, expressed reluctance to accept the HPV vaccine Belief in faith healing and traditional medicine over orthodox methods. A concern that immunizing adolescents might encourage sexual activities, reflecting the cultural and religious expectations of chastity before marriage	
Nigeria, 2020, Nguyen et al. [16]	Cross-sectional Survey 137 healthcare workers from Anambra State	Investigate perceived barriers to HPV vaccination in Nigeria, focusing on health workers’ perceptions	Perceived barriers to HPV vaccination	Lack of awareness Availability and cost as significant barriers to HPV vaccination Female health workers demonstrated less knowledge about HPV and its vaccination than their male counterparts	Most respondents (97%) identified as Christian in a region where cultural and religious leanings influence medical decisions	While religious beliefs were identified as one of the barriers, the study predominantly focused on Christian demographics and did not thoroughly examine the impact of religious diversity on vaccination acceptance
Nigeria, 2021, Okunowo et al. [50]	Cross-sectional Structured questionnaire 208 women from Lagos, Nigeria	Explore predictors, motivators, and barriers to HPV vaccination and testing among urban women	HPV vaccination and testing	HPV vaccination uptake (29.0%) Being employed and unmarried were predictors of HPV vaccination uptake Major motivators: healthcare providers’ recommendations, friends/relatives, and media Significant barriers were fear of the vaccine, lack of awareness, and inaccessibility	Recommendations by religious bodies were one of the motivators and barriers to HPV vaccination and testing.	
Nigeria, 2022, Balogun et al. [30]	Discrete choice experiment Survey 700 participants in Ibadan, Southwest Nigeria	Explore preferences for HPV vaccination among participants in Ibadan, Nigeria	Preferences for attributes of HPV vaccine	Variability in preferences for HPV vaccine attributes Stronger preferences for vaccines with higher efficacy, fewer side effects, and lower costs 35.4% males and 64.5% females	The significant role of religious leaders, with participants’ preferences being shaped not only by practical considerations but also by underlying religious beliefs	
Nigeria, 2023, Elebiyo, [46]	Cross-sectional study Self-administered questionnaire 302 parents at University of Benin Teaching Hospital	Examine knowledge, attitudes, and uptake of HPV vaccine among parents of adolescents	Parental knowledge, attitudes, and uptake of HPV vaccine	Low knowledge of HPV and its vaccine among parents Low uptake of HPV vaccine Moderate approval rate for child immunization	Parents’ religious beliefs significantly influenced their decision to vaccinate their children Those whose religion did not disapprove of vaccination were more likely to vaccinate their children	
Nigeria, 2023, Rabiu & Yahuza, [51]	Cross-sectional study Method not specified 400 students attending two secondary schools in Kano State, Nigeria	Assess knowledge and attitudes regarding HPV infection among secondary school students in Kano State and assess their opinions on HPV vaccination	Knowledge and attitudes towards HPV infection, vaccines, and cervical cancer prevention	Limited knowledge about HPV and cervical cancer among students None of the respondents were vaccinated against HPV 81% were not ready to take the vaccine	Religious beliefs may influence perceptions and decisions regarding HPV vaccination	Religious objections included opinions about the vaccine’s compatibility with religious beliefs, lack of religious endorsement, and concerns about vaccine purity and safety Misconceptions about the causes and transmission of HPV and cervical cancer
Pakistan, 2023, Awan et al. [56]	Cross-sectional study Online questionnaire 1620 participants across various regions of Pakistan	The study explored knowledge, attitudes, and practices related to HPV and anal cancer	Knowledge, attitudes, and practices related to HPV and anal cancer	Low awareness of HPV and anal cancer High stigma and embarrassment regarding screening Strong moral beliefs and minimal engagement in health services and programs Males are more likely than females to support the HPV vaccine	The high percentage of respondents practicing moral beliefs (89%) indicate that religious or cultural beliefs play a role in their attitudes towards HPV Most participants were Muslim (99.3%), which might imply that Islamic beliefs could have influenced their attitudes and practices	Myths or religious beliefs could prevent individuals from HPV vaccination (71% agreed)
Saudi Arabia, 2019, Jradi & Bawazir [48]	Qualitative study Focus groups and in-depth interviews 135 women in Riyadh City	Examine awareness of HPV and attitudes towards the vaccine among Saudi women	Awareness and attitudes towards HPV and the vaccine	Cultural barriers Lack of awareness about HPV and cervical cancer	Cultural factors and religious beliefs are often intertwined, significantly influencing health-related decisions.	Concerns about the vaccine’s necessity and safety, along with fears that it might promote promiscuity Vaccine’s compatibility with conservative societal values Cultural resistance to HPV vaccination, particularly due to concerns about promoting promiscuity
Saudi Arabia, 2022, Darraj et al. [28]	Cross-sectional study Survey 569 adults in Jazan Province	Assess the level of knowledge of HPV and acceptability of the vaccine among adults in Jazan Province, Saudi Arabia	Knowledge and attitudes towards HPV and vaccination	Low level of HPV knowledge and varying degrees of vaccine acceptability Gender analysis showed no significant difference in knowledge or acceptability	30% opposed the vaccine for religious reasons Religious practice is intricately linked to vaccine attitudes	Religious objections influence attitudes toward HPV vaccination
Saudi Arabia, 2023, Abdelaliem et al. [45]	Cross-sectional study Self-administered online survey 307 College of Nursing students	Predict knowledge and attitudes regarding HPV and its vaccine among nursing students	Knowledge and attitudes toward HPV and vaccination	Low knowledge of HPV Moderately positive attitudes		About 30% of the participants opposed HPV vaccination for religious reasons.
Uganda, 2017, Turiho et al. [52]	Qualitative study Focus group discussions and key informant interviews Ten participants (three health workers, two community leaders, five teachers) from Ibanda district, Western Uganda	Explore community members’ perceptions about HPV vaccination and the implications of these perceptions for acceptance	Perceptions and acceptability of HPV vaccination	Varied perceptions of HPV vaccination, with both acceptance and concerns impacting its acceptability	Fears about HPV vaccination may be influenced or amplified by religious beliefs, even if not directly cited as such	Fears about the vaccine causing infertility or being a form of population control, possibly tied to religious beliefs about the sanctity of life and procreation Rumors about the vaccine causing diseases or being experimental, which could be fueled by skepticism about modern medicine
Uganda, 2023, Odongo Ojok et al. [49]	Cross-sectional study Community-based survey using a semi-structured questionnaire 197 girls living in the Pece-Laroo Division	Assess HPV vaccine coverage and factors influencing its utilization among girls aged 9–13 years in the Gulu District, Uganda	HPV vaccine coverage and utilization	Low vaccine use (35%) influenced by knowledge of HPV, methods of prevention, and mobilization efforts	87.3% of the respondents agreed that the vaccine was not against their religious beliefs, indicating a general acceptance of the vaccine	High acceptance of the HPV vaccine in relation to their religious beliefs The majority did not perceive the vaccine as conflicting with their religious values
United Arab Emirates, 2013, Ortashi et al. [42]	Cross-sectional survey Self-administered questionnaire 356 male students from different universities in the UAE	Assess knowledge about and acceptability of HPV vaccination among male university students	Knowledge and acceptability of HPV vaccination	Low knowledge of HPV among students (32%) Only 46% would accept vaccination Concerns about vaccine safety and objections from a religious authority (25%)		Objections from a religious authority were noted as a barrier to vaccine uptake by 25% of participants Despite the objections, a significant number of students were open to vaccination, indicating a varied interpretation of religious beliefs in the context of health decisions
United Arab Emirates, 2022, Al Shdefat et al. [44]	Cross-sectional Questionnaire 400 Emirati men	To determine Emirati men’s opinions about the HPV vaccination, specifically whether they would use it themselves or allow their female relatives to use it	HPV vaccine acceptability	37% acceptance rate for HPV vaccine among Emirati men, with cultural and safety concerns 20.5% would recommend the vaccine if they believed it to be safe 22.6% would not recommend it, even if considered safe	Almost half (41%) of the respondents would not recommend the vaccine even if it was recommended by a religious authority.	Religious objections are one of the barriers to the wide introduction of the HPV vaccine Among the respondents, 2.1% stated the vaccine was religiously unacceptable

### Religious beliefs and practices on HPV vaccine acceptance

The findings of this review highlight the complex interplay between religious beliefs and HPV vaccine acceptance among various populations. Some studies have indicated that religious beliefs did not influence vaccine uptake [40,41,43,44,49], while others found that religious beliefs had the opposite effect [21,28,45,47,51,55–57]. This influence stemmed from various beliefs, including “health outcomes being determined by God’s will” [21], faith healing and traditional medicine over orthodox methods [22], religious beliefs influencing health-related decisions [16,47,48], trust in religious bodies [30,50], parents with religious beliefs [53], or need for their husbands’ approval for vaccination [54]. Elebiyo (2023) showed that Nigerian parents’ religious beliefs significantly influenced their decision to vaccinate their children [46]. Concerns about the HPV vaccine being seen as a license for sexual promiscuity reflected cultural and religious expectations around chastity before marriage in Nigeria [22]. Conversely, Ling et al. (2012) [40] found a high acceptance of HPV vaccination among teachers in a predominantly Islamic country, challenging the notion that religion is inherently anti-vaccination.

### Religious objections and misconceptions

This review identified several misconceptions and objections about HPV vaccination that are rooted in religious beliefs. These misconceptions include the belief that the vaccine causes diseases, is unnecessary in certain situations, or is still experimental, as well as religious skepticism about modern medicine [52]. In addition, there is a perception that the vaccine may serve as a license for unbridled sex, reflecting religious expectations about chastity before marriage [48,51]. Some hold beliefs about vaccines containing haram substances and being unnatural [43]. There is also a perception that the vaccine is unnecessary for married couples who enjoy a proper religious upbringing [54]. There are also worries about the vaccine causing infertility [52,57]. Additionally, there are questions about the vaccine’s compatibility with religious beliefs and lack of religious endorsement [51], while some see the HPV vaccine as a surreptitious form of birth control [57]. Objections from religious authorities were reported in various studies [22,28,42]. In contrast, one study using a community-based survey among girls reported no religious misconceptions about the HPV vaccine [49].

### Suggestions for public health interventions

Some of the studies also suggested public health strategies to increase uptake by increasing awareness among people refusing the HPV vaccine about religious beliefs and misconceptions about HPV vaccination. Some studies recommended public health campaigns that address religious concerns via scientific information [21,28,41,44,46,49,53–56]. Other studies suggested collaborating with religious leaders and institutions to influence community attitudes on health-related decisions [16,22,40] and develop culturally and religiously sensitive educational materials to bridge the gap between scientific knowledge and religious understanding, or utilize religious platforms for health education [42,43,52,57]. Public health officials need to be sensitive to religious objections, addressing them respectfully and informally [48,50].

## Discussion

By relying on the relevant literature, this review aimed to understand the religious factors that play a role in HPV vaccine acceptance and decision-making in Islamic countriesThe review incorporated studies from various cultural and religious settings. The explored concepts centered on the interplay of religious beliefs, health, and vaccine acceptance. Most of the studies focused on awareness, attitudes, and acceptance of the vaccine among groups such as university students, parents, healthcare providers, and school nurses. The studies investigated the factors that influence vaccination decisions but did not explore how religious beliefs play a role in the decision to vaccinate. Thus, this review included only those studies that presented variations in religious beliefs related to HPV vaccination and its acceptability in Islamic countries.

During the COVID-19 pandemic, an important issue that emerged was the public’s trust in vaccination programs in general. While this review focused on studies published up to October 2023, it is notable that not one of them addressed the intersection of COVID-19 with HPV vaccine perceptions. The global health crisis has undeniably influenced public attitudes towards vaccines. For instance, increased vaccine hesitancy or, conversely, a heightened trust in the power of vaccines due to the rapid development of COVID-19 vaccines could influence public perception of other remedies, including those for HPV [58]. This intersection presents a unique opportunity to examine how crises impact long-term vaccine strategies and acceptance, particularly in contexts where vaccine hesitancy is already influenced by complex factors such as religious beliefs and cultural practices [21,41,58]. Future research could include comparative analyses of HPV vaccine acceptance pre- and post-COVID to better understand the pandemic’s impact on public trust and vaccination behavior. Public health campaigns promoting the HPV vaccine should consider incorporating lessons learned from the COVID vaccination efforts. This includes addressing misinformation, leveraging trusted community and religious leaders, and ensuring transparent communication about vaccine safety and efficacy [16,48].

The review also found that the degree of religious influence on health decisions varies not only between countries but also within different regions of the same country, reflecting the diversity in religious denominations and sects [28,45]. These findings are consistent with previous studies in non-Islamic countries [18,23,59]. Religious affiliation influenced acceptability, showing fewer acceptors among Hindus and Muslims than those without religious affiliation [60]. However, more recently, Coleman and colleagues (2024) reported that religiosity had little effect on HPV vaccine decisions for urban, Black, and Hispanic parents [61].

Best and colleagues (2019) proved with further analysis that the relationship between religious beliefs and HPV vaccination was fully mediated by sexual activity [18]. This finding may clarify the link between religious beliefs and vaccine acceptance because strong religious or spiritual beliefs are often associated with abstaining from premarital sex. Given that sexually active individuals are at higher risk for HPV infection, the decision to get vaccinated is tied to one’s faith and lifestyle [23]. This also explains the doubts about the vaccine’s necessity, another finding of the present review.

Studies showed that a common question within Islamic countries is why a pious family with sexually inactive children needs to be immunized against sexually transmissible diseases at all. Commitment to religious principles, such as abstaining from premarital sex, was frequently indicated in the included studies. This finding is important because it aligns with the belief that having sex outside of marriage is a sin and that adhering to religious principles obviates the need for HPV vaccination [62,63]. This finding resonates with studies conducted in various religious contexts. For instance, a survey of 1557 Christian college students in the USA identified that following the injunction against premarital sex is the greatest predictor of HPV vaccine uptake [59]. Another study on Christian teachings about sexual relationships among parents found that religious propriety had a negative impact on the intention to vaccinate children [64]. A qualitative study of Jewish mothers stated that the religious laws governing family purity and abstinence until marriage are the reasons for their daughters’ low HPV vaccine uptake [29].

One noteworthy finding in this review was the belief that the HPV vaccine would encourage wanton sexual behavior. This sentiment was particularly pronounced in regions where conservative religious beliefs strongly influence societal norms [60]. Other studies on HPV-related beliefs and vaccine acceptability in the USA and Kenya revealed that parents were concerned that vaccination might encourage sexual activity at a younger age, elevate risky behaviors, and contribute to increased promiscuity [65,66]. There are contradictory studies in the literature about whether these fears are justified. Some studies found no connection between HPV vaccination status and age of sexual onset or number of sexual partners [67,68]. On the other hand, Brabin and colleagues (2009) reported that HPV-vaccinated girls aged 12–13 years stated that they might engage in more risky sex after vaccination [69]. These findings underscore the complex interplay between cultural beliefs and public health interventions, highlighting the need for culturally sensitive education and communication strategies to address misconceptions about the HPV vaccine.

Some individuals perceive health as beyond their control, surrendering themselves to luck, fate, or a higher power [70]. In this review, parents’ religious beliefs were found to influence HPV vaccine acceptance. This result is consistent with previous studies [11,12,32]. Parents who strongly adhere to religious or cultural views are less likely to accept HPV vaccination [60]. A qualitative study involving parents of adolescents from Arabic backgrounds in Western Sydney revealed the role of parents’ religion in forming attitudes about HPV vaccination [63]. Children born to Muslim mothers were found to have a higher likelihood of being under- or unvaccinated compared to their Hindu counterparts [33]. Many female students in the French-speaking part of Switzerland often cited parental opposition as one of the primary reasons for declining HPV vaccination [11].

Moreover, the review identified misconceptions about the vaccine’s composition. This finding is consistent with those of a study that some Muslim students believe the vaccine contains pig protein, which is why Muslim families may avoid using it [59]. Many mothers indicated that they would not permit their teenage children to have the HPV vaccine if it was non-halal [63]. This result also explains why some religious leaders are against vaccination. A study revealed that some imams have forbidden the use of vaccines due to their alleged porcine components [71]. Islamic law prohibits using medicines or ingredients derived from haram sources, specifically those containing pig and its derivatives [72].

Despite considerable evidence showing little connection between the HPV vaccine and infertility, rumors about the vaccines serving some genocidal purpose continue in the Islamic community. This review highlighted the paucity of qualitative studies addressing the association between the HPV vaccine and infertility in Islamic countries. Studies in Islamic countries have mainly focused on concerns about vaccines in general. For example, in a study by Sheikh and colleagues (2013) [71], participants stated that “Vaccination is a conspiracy of the Zionists. Vaccinating our children will inevitably make them sterile.” Schuler and colleagues (2014) [73] reported that mothers with concerns about vaccine-associated infertility had less intention to vaccinate their sons than other parents. And yet, a self-reported survey of women aged 20 to 33 showed that those who had been married and had received an HPV vaccine were less likely to report infertility [74]. A recent systematic review and meta-analysis study shed light on the need for high-quality prospective studies to confirm the relationship between the HPV vaccine and infertility [75].

Objections were observed in the studies that examined the role of religious authorities in shaping attitudes towards HPV vaccination. These objections may have arisen from concerns about the vaccine’s compatibility with religious principles or broader ethical considerations, emphasizing the need for a nuanced engagement with religious leaders to promote informed decision-making regarding vaccination. A study from South Dakota found that religious leaders’ messages were more effective than statements from political or medical figures in shaping a positive perception of the COVID-19 vaccine [76]. Conflicts between religious practices and medical recommendations can lead to misunderstandings and poor treatment adherence [70]. This finding emphasizes the impact of religious leaders on health-related behaviors within their communities. The role played by religious leaders or institutions suggests that collaborating with them could enhance health education for HPV vaccine acceptance. Therefore, recognizing the importance of diversity is vital for understanding specific religious dynamics and ensuring that public health strategies remain culturally and religiously sensitive.

## Limitations

This scoping review has several limitations. First, its geographical focus is primarily on the OIC countries. This may limit the findings’ applicability to other regions with different religious and cultural backgrounds. Second, the literature search was limited to three databases: MEDLINE/PubMed, Embase, and CINAHL. This selection might exclude relevant studies published in local journals or languages other than English. Third, the methodological diversity among the included studies, encompassing various research designs and sample sizes, complicates data comparison and synthesis, potentially impacting the conclusion’s strengths. Fourth, focusing on religious factors might lead to underestimating other important determinants such as socioeconomic status, education, and healthcare access, which can also influence health decisions and vaccine uptake. Fifth, the review did not find any articles that specifically compared HPV vaccine acceptance between the Sunni and Shia branches of Islam. Although most of the countries in this review are predominantly Sunni, the lack of explicit comparisons limits our understanding of potential differences in religious influence on vaccine acceptance. The primary aim of this study was to explore the broader impact of religious beliefs on HPV vaccine acceptance within the OIC countries. The existing literature did not provide explicit comparisons between these branches. Future research should explore these potential differences to provide a more nuanced understanding of the impact of religious beliefs on HPV vaccination. Sixth, the majority of the included studies did not conduct gender-specific analyses, which limits our ability to fully understand gender differences in HPV vaccine acceptance across different cultural contexts. Seventh, none of the included studies explicitly addressed the impact of COVID-19 on HPV vaccine perceptions. The pandemic has significantly influenced public perceptions of vaccines, highlighting issues of vaccine hesitancy, misinformation, and public trust. Future research should include comparative analyses of HPV vaccine acceptance pre- and post-COVID to understand the pandemic’s impact on vaccination behavior and public trust. Lastly, despite a systematic approach, the restriction to English language studies and Muslim countries could lead to favoring certain types of studies, possibly overlooking useful research that does not fit the predefined parameters. Future research should employ multilingual searches to ensure a more comprehensive inclusion of relevant literature.

## Conclusion

Several findings from this study contribute to understanding the religious factors that influence the acceptance of HPV vaccines. The review showed that religious beliefs did not always affect overall vaccine uptake, but they did influence vaccine acceptability. The study identified a range of misconceptions and beliefs related to HPV vaccination. Some of these misconceptions included seeing the vaccine as a form of ethnic cleansing, a license for wanton behavior, a defiance of religious norms, a sneaky way to inject good Muslims with haram ingredients, and an abandonment of righteous principles in general.

This study also investigated public health interventions that responded to these misconceptions. To encourage HPV vaccine uptake in Islamic countries, public health strategies must adopt a holistic approach that incorporates religious, cultural, and social aspects. A key strategy mentioned in the studies is engagement with religious leaders and communities. Leveraging the influence of religious leaders can shift community attitudes toward vaccine acceptance, especially when the messages are aligned with religious teachings and values. Educational materials should present the facts about HPV vaccines in a manner that respects religious beliefs. The adaptability of public health campaigns to regional variations in faith and practices can ensure that the interventions are relevant to different cultural contexts. Training health professionals in religious literacy and cultural competence is also essential,because it can equip them to better understand and address the concerns of the communities they serve. Future research should focus on deepening our understanding of the dynamic relationship between religious beliefs and health behaviors. Comparative studies across different contexts are essential for understanding the different ways that religion influences health decisions. Qualitative studies are critical as they can provide a deeper understanding of religious beliefs on vaccine acceptance and other religious-related health behaviors.

## Supporting information

S1 Table PRISMA-Scr 2020 checklist. (DOCX)
